# Screening of characteristic genes in ulcerative colitis by integrating gene expression profiles

**DOI:** 10.1186/s12876-021-01940-0

**Published:** 2021-10-30

**Authors:** Yingbo Han, Xiumin Liu, Hongmei Dong, Dacheng Wen

**Affiliations:** 1 Department of Gastrointestinal Nutrition and Hernia Surgery, The Second Hospital of Jilin University, Nanguan District, Number 218, Ziqiang Street, Changchun, 130041 China; 2 Department of Clinical Laboratory, The Second Hospital of Jilin University, Number 218, Ziqiang Street, Nanguan District, Changchun, 130041 China

**Keywords:** Ulcerative colitis, Weighted gene coexpression network analysis, Feature genes, Protein–protein interaction

## Abstract

**Background:**

This study aimed to screen the feature modules and characteristic genes related to ulcerative colitis (UC) and construct a support vector machine (SVM) classifier to distinguish UC patients.

**Methods:**

Four datasets that contained UC and control samples were obtained from the Gene Expression Omnibus database. Differentially expressed genes (DEGs) with consistency were screened via the MetaDE method. The weighted gene coexpression network (WGCNA) was used to distinguish significant modules based on the four datasets. The protein–protein interaction network was established based on intersection genes. Enrichment analysis of Gene Ontology (GO) biological processes (BPs) and Kyoto Encyclopedia of Genes and Genomes (KEGG) pathway enrichment were established based on DAVID. An SVM combined with recursive feature elimination was also applied to construct a disease classifier for the disease diagnosis of UC patients. The efficacy of the SVM classifier was evaluated through receiver operating characteristic curves.

**Results:**

Twelve highly preserved modules were obtained using the WGCNA, and 2009 DEGs with significant consistency were selected using the MetaDE method. Sixteen significantly related GO BPs and 12 KEGG pathways were obtained, such as cytokine-cytokine receptor interaction, cell adhesion molecules, and leukocyte transendothelial migration. Subsequently, 41 genes were used to construct an SVM classifier, such as *CXCL1*, *CCR2*, *IL1B*, and *IL1A.* The area under the curve (AUC) was 0.999 in the training dataset, whereas the AUC was 0.886, 0.790, and 0.819 in the validation set (GSE65114, GSE37283, and GSE36807, respectively).

**Conclusions:**

An SVM classifier based on feature genes might correctly identify healthy people or UC patients.

**Supplementary Information:**

The online version contains supplementary material available at 10.1186/s12876-021-01940-0.

## Background

Ulcerative colitis (UC) is an inflammatory intestinal disease characterized by easy recurrence and chronic persistence [1]. The lesion site is mainly confined to the large intestine mucosa and submucosa. Mucosal inflammation at the onset site has diffuse distribution and extends to the rectum. The main clinical manifestations are abdominal pain, diarrhea, and mucinous pus bloody stool. It is easy to cause intestinal fibrosis and increase colon cancer risk [2]. Medical circles at home and abroad hope to make achievements in UC treatment, but its etiology is still unclear and its pathogenesis is complex, so it is listed as one of the modern refractory diseases by the World Health Organization [3, 4].

With the development of high-throughput microarray technology, the identification of genomic variation has been promoted, which will help to understand the pathogenesis of potential biomarkers in many diseases [5]. Kang et al. [6] reported that the diagnosis of UC is usually delayed, but the relationship between delayed diagnosis and prognosis of UC has not been widely studied. Biasci et al. [3] reported that genes from the best classifiers are optimized by quantitative polymerase chain reaction (qPCR) and the best qPCR classifier is distinguished using further machine learning, which could evaluate the prognosis of newly diagnosed UC patients. In previous years, research has identified many molecular markers that could help in the early diagnosis of UC. For example, Zhang et al. [7] reported that *IL6*, *PTPRC*, *CXCL8*, *IL1B*, and *MMP9* might be the key genes that could provide vital markers for the early diagnosis and treatment for UC. Zhu et al. [8] found several genes associated with the development of UC, such as *MMP1*, *REG1A*, and *AQP8*. Yan et al. [9] found 11 mutated genes differentially expressed in UC samples, such as *APC*, *APOB*, *MECP2*, *NCOR2*, and *USP48*. All these reports suggested that feature genes might play an important role in the diagnosis of UC.

Thus, in this study, the weighted gene coexpression network (WGCNA) was used to distinguish stable modules from four datasets. Then, the protein–protein interaction (PPI) network was constructed through differentially expressed genes (DEGs) in stable modules. A support vector machine (SVM) combined with recursive feature elimination (RFE) was also applied to construct a disease classifier for the disease diagnosis of UC patients.

## Methods

### Screening of expression profile data

“Ulcerative colitis” and “Human” were used as keywords to search all publicly uploaded expression profile data from the National Center for Biotechnology Information Gene Expression Omnibus database (http://www.ncbi.nlm.nih.gov/geo/). This study contained four datasets: GSE65114 (n = 28; 16 UC and 12 control), GSE36807 (n = 22; 15 UC and 7 control), GSE37283 (n = 20; 15 UC and 5 control), and GSE59071 (n = 108; 97 UC and 11 control). The selection standards of datasets were as follows: [1] the dataset was a gene expression profile, [2] the samples are solid samples of intestinal tissue from UC patients, and [3] the samples contained control samples. Microarray raw data (GPL570, CEL files) from the four datasets were obtained from the Affymetrix platform (Santa Clara, CA, USA), which were processed for background correction (MAS) and quantile normalization using Affy package in R3.4.1 version 1.60.6 (http://www.bioconductor.org/packages/release/bioc/html/affy.html) [10].

### Meta-analysis of DEGs

Unlike methods of screening DEGs in previous studies, the meta-synthesis algorithm was adopted to screen DEGs with consistency in multiple datasets. The meta-analysis aimed to cite multiple studies, collect multiple experimental datasets, and screen for reliable genes. However, the four datasets in this study were obtained from different patient samples and experimental detection. There may be different degrees of bias in the dataset; thus, MetaQC (https://cran.r-project.org/web/packages/MetaQC/index.html) was first used to carry out objective quality-control on the datasets combined with principal component analysis (PCA) two-dimensional map and standardized mean rank to evaluate and screen datasets.

DEGs were then screened by MetaDE.ES in the MetaDE package (https://cran.r-project.org/web/packages/MetaDE) [11]. To evaluate gene expression consistency, the heterogeneity of the four datasets was checked through the τ^2^, Q value, and Qpval values (judgment criteria: when the value of the statistic τ^2^ is 0), it indicates that each research object is homogeneous and unbiased; the statistic Q obeys the χ^2^ test with a degree of freedom of k-1; when Qpval > 0.05, it indicates that each research object is homogeneous and unbiased). τ^2^ = 0 and Qpval > 0.05 were selected as homogeneity test parameters, whereas false discover rate (FDR) < 0.05 was as the threshold for differential gene expression. DEGs with consistency in different groups were detected using the MetaDE method with the cutoff criterion of *p* < 0.05. FDR was obtained through multiple test corrections. FDR < 0.05 indicated a significant difference. Each individual dataset was calculated separately to express the fold change. Genes were kept consistent by combining with log_2_FC orientation in each dataset.

### Significant gene module based on the WGCNA

In this study, GSE59071 was used as the training dataset and GSE65114, GSE36807, and GSE37283 were used as the validation datasets. WGCNA package version 1.61 (https://cran.r-project.org/web/packages/WGCNA/index.html) [12] in R3.4.1 was applied to select the significant gene module associated with UC in the four datasets. The WGCNA algorithm is implemented according to the steps of defining adjacency function, dividing gene modules, and evaluating module stability. The threshold of gene module partition and screening is that the gene module contains at least 100 genes with a cut height of 0.995. Genes in the important modules were selected as the object for further analysis.

Screened DEGs were mapped to each WGCNA module to calculate the fold enrichment ratio and the p value of target DEGs in each WGCNA module using the hypergeometric algorithm. The formula is f(k,N,M,n) = C(k,M) * C(n-k,N-M) / C(n,N) [13], where N represents all genes referred to the analysis of the WGCNA algorithm, M is the gene number in each module obtained by the WGCNA algorithm, n is the number of obvious DEGs, and k is the DEG number mapped to the corresponding modules. The significant enrichment parameters fold enrichment and enrichment significance p values of the target significant DEGs were calculated in each WGCNA module. The threshold was set as *p* < 0.05 and fold enrichment > 1. Finally, DEGs markedly enriched in the stable WGCNA module obtained from the screening were compared to consistent and significant DEGs selected in the previous step, and the intersection part was taken. Enrichment analysis of Gene Ontology (GO) biological processes (BPs) and Kyoto Encyclopedia of Genes and Genomes (KEGG) pathway enrichment were established based on DAVID version 6.8 (https://david.ncifcrf.gov/) [14, 15] with a cutoff of *p* < 0.05.

### PPI network construction

STRING version 10.5 (https://string-db.org/) [16] was applied to search for the interaction between gene products and proteins used for constructing the PPI network. The gene interaction network was visualized through Cytoscape version 3.6.1 (http://www.cytoscape.org/) [17]. The KEGG pathways of DEGs that constitute the interaction network were analyzed based on DAVID.

### Screening of key genes related to UC

All KEGG pathways associated with UC were searched from the Comparative Toxicogenomics Database 2019 update (http://ctd.mdibl.org/) [18], which were compared to the pathways in the PPI network. A PPI network of KEGG pathways directly related to UC and screened key genes involved in the UC pathways was constructed.

### Construction of the sample-type recognition classifier

In the GSE59071 training dataset, the RFE, R3.4.1 caret package version 6.0–76 (https://cran.r-project.org/web/packages/caret) [19, 20], was applied to screen optimized feature gene combinations. The gene combination with the highest sample recognition accuracy in 100-fold cross-validation was selected as a feature gene combination [21]. The SVM [21] function in the e1071 package of R3.4.1 version 1.6–8 (https://cran.r-project.org/web/packages/e1071) was used to establish the SVM classifier for further analysis. The effectiveness of the SVM classifier was evaluated in the training dataset and three validation datasets. pROC package version 1.12.1 (https://cran.r-project.org/web/packages/pROC/index.html) [22] in R3.4.1 was applied to obtain sensitivity, specificity [23], and area under the curve (AUC) [24].

## Results

### Screening of DEGs with significant consistency by meta-analysis

**Fig. 1:**
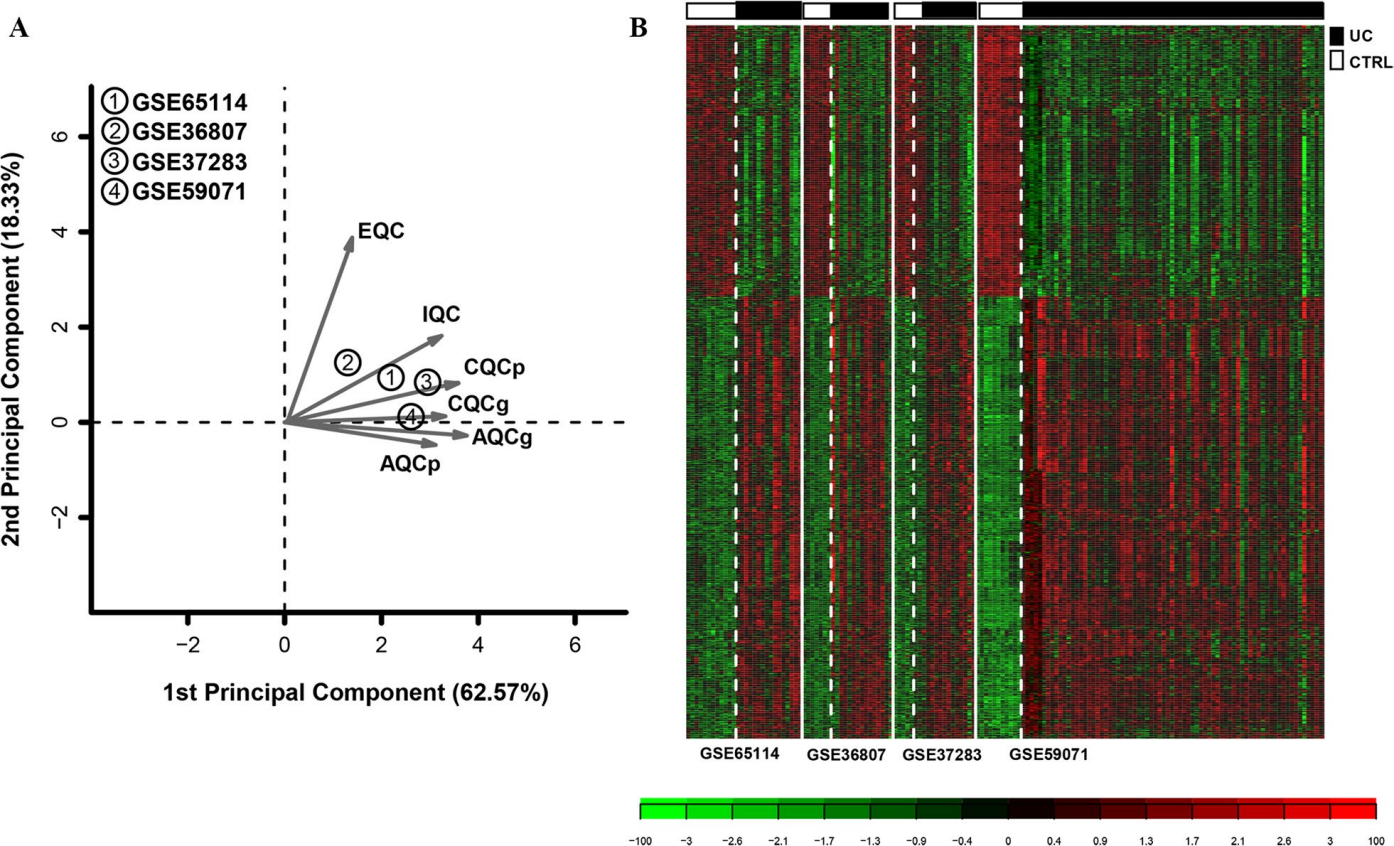
(**A**) PCA principal component plan. MetaQC quality-control chart of GSE65114, GSE36807, GSE37283, and GSE59071 datasets. Horizontal and vertical axes indicate the first and second principal components in the PCA, respectively, whereas 1 to 4 represent the four corresponding datasets. (**B**) Heatmap of DEGs with significant consistency

First, we perform data standardization on each of the 4 datasets, which was shown in Additional file 1: Table 1. A total of 16,337 genes were obtained from the four datasets. MetaQC quality-control inspection was conducted on the four datasets (Table 1). The PCA principal component plan is shown in Fig. 1A, indicating that the four datasets are evenly distributed. The sum of PC1 and PC2 was 80.90%, and these indexes met the data quality testing standards that should be included in subsequent analysis. Subsequently, 2009 DEGs with obvious consistency were screened from the four datasets for the next analysis (Additional file 2: Table 2). In Fig. 1B, DEGs screened from four different datasets were consistent in the degree of difference and direction of maladjustment.

**Table 1:** The information in GSE65114, GSE36807, GSE37283 and GSE59071 datasets

ID	Platform	Total sample number	UC	CTRL
GSE65114	GPL16686	28	16	12
GSE36807	GPL570	22	15	7
GSE37283	GPL13158	20	15	5
GSE59071	GPL6244	108	97	11

UC: Ulcerative colitis; CTRL: control

**Table 2:** Preservation information of nine co-expression modules in GSE65114, GSE36807, GSE37283 and GSE59071 datasets

ID	Color	Module size	Preservation infor		#DEGs	Enrichment infor	
			Z-score	*p* Value		Enrichment fold [95%CI]	P_hyper_
Module 1	Black	266	10.0865	5.10E−14	78	1.468 [1.116–1.914]	4.97E−03
Module 2	Blue	441	18.1473	8.50E−27	116	1.317 [1.053–1.637]	1.34E−02
Module 3	Brown	433	11.0851	3.50E−13	115	1.329 [1.062–1.655]	1.08E−02
Module 4	Green	386	11.0543	7.30E−46	40	0.519 [0.363–0.725]	4.20E−05
Module 5	Greenyellow	105	0.3610	7.40E−02	14	0.668 [0.351–1.177]	1.72E−01
Module 6	Grey	2037	8.0314	1.00E−200	259	0.637 [0.549–0.737]	3.47E−10
Module 7	Magenta	130	2.2262	4.10E−03	17	0.655 [0.368–1.095]	1.15E−01
Module 8	Pink	170	1.9442	1.60E−03	15	0.442 [0.241–0.753]	1.19E−03
Module 9	Purple	115	5.0647	1.60E−06	3	0.131 [0.0265–0.392]	2.07E−06
Module 10	Red	278	14.0746	2.60E−13	100	1.801 [1.405–2.294]	3.17E−06
Module 11	Turquoise	585	12.9525	2.50E−38	145	1.241 [1.016–1.509]	3.28E−02
Module 12	Yellow	392	15.9048	1.10E−15	164	2.095 [1.715–2.551]	8.57E−13

DEGs: differentially expressed genes

### Identification of vital WGCNA modules

**Fig. 2:**
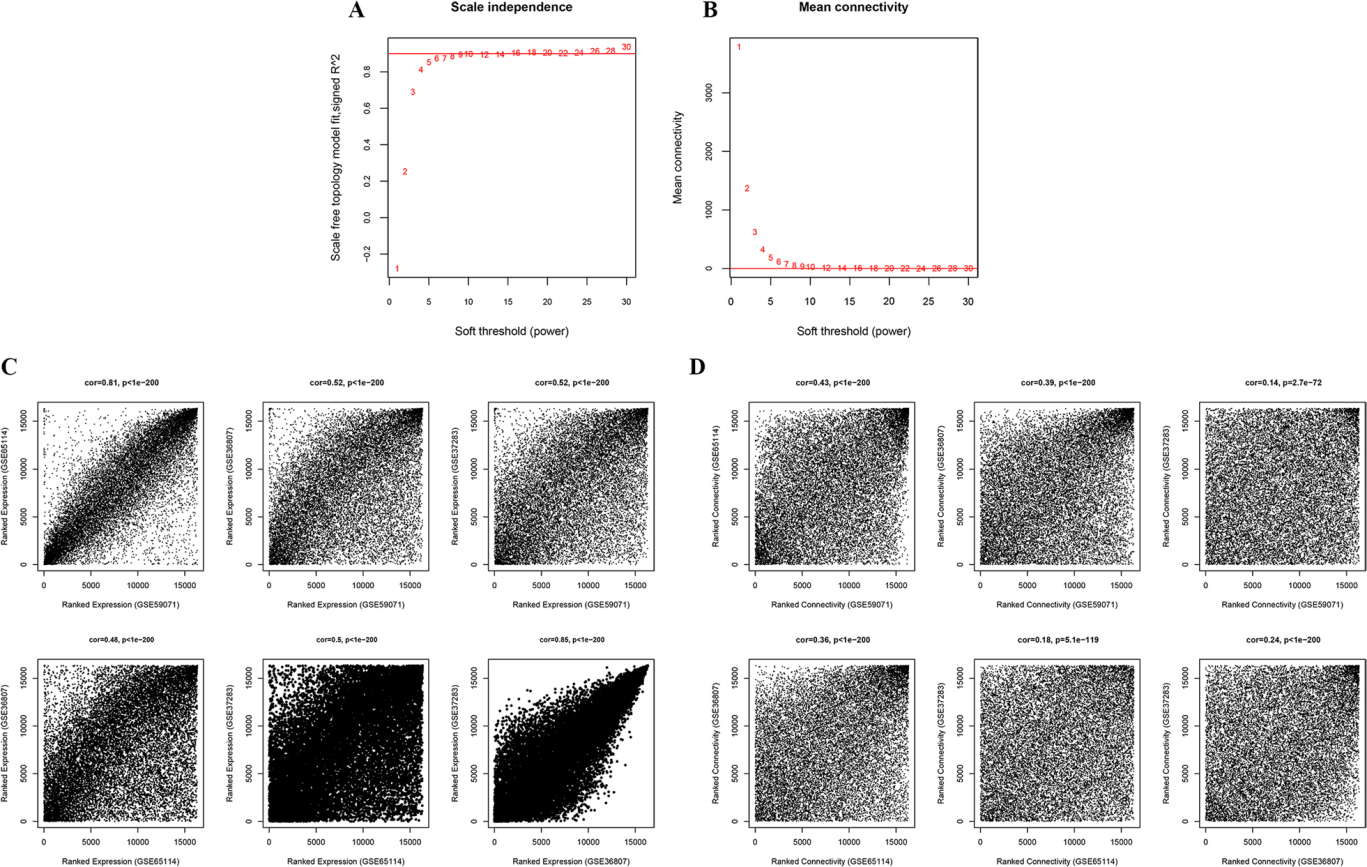
(**A**) Power selection graph of the adjacency matrix weight parameter. The horizontal axis indicates the weight parameter power; the vertical axis indicates the square of the correlation coefficient between log(k) and log[p(k)] in the corresponding network. The red line represents the standard line where the square of the correlation coefficient reaches 0.9. (**B**) Schematic diagram of the average gene connectivity under different power parameters. The red line represents the value of the average connectivity of network nodes under the power parameter value of the adjacency matrix weight parameter in (**A**). (**C**) Correlation graph of the expression levels on the training and validation datasets. (**D**) Training and validation dataset node connection correlation graph

This study aimed to distinguish UC-related modules through the WGCNA. GSE59071 was the training dataset, whereas GSE65114, GSE36807, and GSE37283 were the validation datasets. Both training and validation datasets had a high positive correlation, and the correlation significance p values were < 0.05, a very significant positive correlation, indicating that the data are comparable (Fig. 2). Gene correlation coefficients were calculated from the four datasets. The correlation coefficients between GSE59071 and GSE65114, GSE36807, and GSE37283 were 0.81, 0.52, and 0.52, with *p* < 1e-200 among the three datasets. These data expressed good homogeneity of DEGs in the four datasets (Additional file 3: Table 3).

**Table 3:** GO functional and KEGG pathway enrichment analysis of 718 overlapping genes

Category	Term	Count	*p* Value	FDR
Biology process	GO:0,006,955 ~ immune response	78	8.620E−18	2.280E−14
	GO:0,006,952 ~ defense response	62	7.320E−12	9.700E−09
	GO:0,006,954 ~ inflammatory response	40	2.470E−10	2.180E−07
	GO:0,009,611 ~ response to wounding	53	4.300E−10	2.850E−07
	GO:0,032,963 ~ collagen metabolic process	11	4.540E−08	2.410E−05
	GO:0,050,865 ~ regulation of cell activation	25	5.980E−08	2.640E−05
	GO:0,042,330 ~ taxis	23	2.040E−07	6.750E−05
	GO:0,006,935 ~ chemotaxis	23	2.040E−07	6.750E−05
	GO:0,044,236 ~ multicellular organismal metabolic process	11	8.820E−07	2.600E−04
	GO:0,051,249 ~ regulation of lymphocyte activation	21	9.650E−07	2.560E−04
	GO:0,002,694 ~ regulation of leukocyte activation	21	5.860E−06	1.412E−03
	GO:0,002,683 ~ negative regulation of immune system process	14	1.550E−05	3.412E−03
	GO:0,007,155 ~ cell adhesion	51	1.690E−05	3.448E−03
	GO:0,022,610 ~ biological adhesion	51	1.730E−05	3.276E−03
	GO:0,006,968 ~ cellular defense response	12	1.780E−05	3.140E−03
	GO:0,050,864 ~ regulation of B cell activation	11	1.990E−05	3.297E−03
KEGG pathway	hsa04514:Cell adhesion molecules (CAMs)	18	2.450E−06	1.990E−04
	hsa04060:Cytokine-cytokine receptor interaction	27	4.490E−06	3.640E−04
	hsa04660:T cell receptor signaling pathway	14	2.590E−05	2.098E−03
	hsa00910:Nitrogen metabolism	6	5.340E−05	4.325E−03
	hsa04670:Leukocyte transendothelial migration	14	5.670E−05	4.592E−03
	hsa04062:Chemokine signaling pathway	18	1.140E−04	9.204E−03
	hsa04512:ECM-receptor interaction	10	2.510E−04	2.031E−02
	hsa04640:Hematopoietic cell lineage	10	2.880E−04	2.333E−02
	hsa04630:Jak-STAT signaling pathway	14	4.730E−04	3.828E−02
	hsa04650:Natural killer cell mediated cytotoxicity	12	5.660E−04	4.588E−02
	hsa04210:Apoptosis	9	5.680E−04	4.604E−02
	hsa04672:Intestinal immune network for IgA production	6	6.030E−04	4.885E−02

**Fig. 3:**
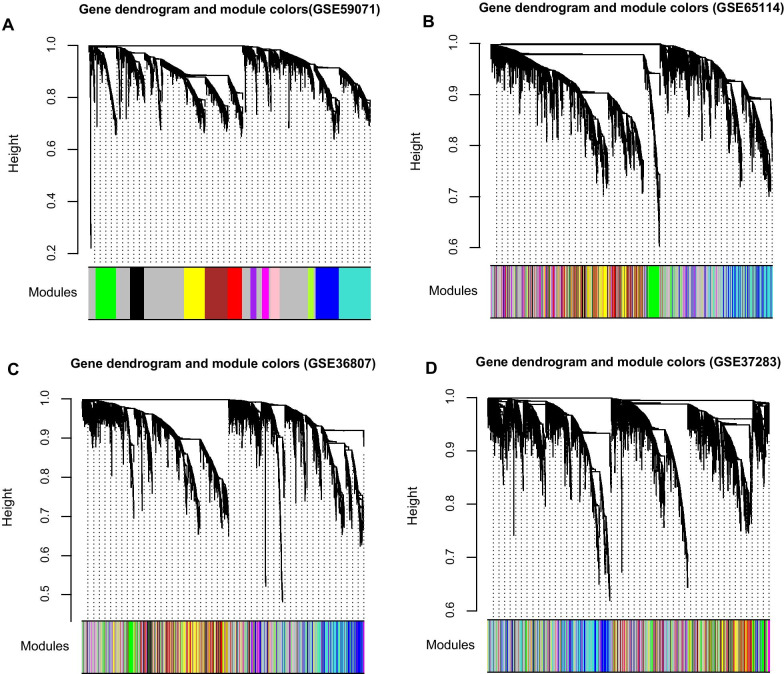
Screening of gene modules in the (**A**) training dataset of GSE59071 and the validation datasets of (**B**) GSE65114, (**C**) GSE36807, and (**D**) GSE37283

Twelve modules were screened through the WGCNA with a cut height of 0.995, and the amount of genes in each gene network was 25 as a criterion (Fig. 3A). Genes were colored based on the module color in the training dataset (Fig. 3).

**Fig. 4:**
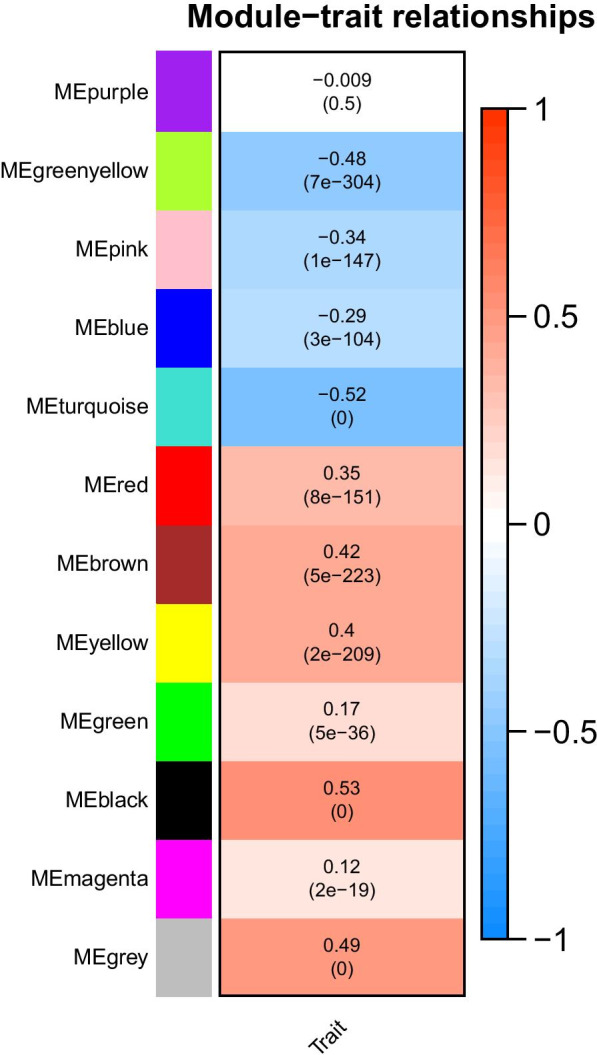
Correlation analysis performed between each stable module and UC characterization

The preservation Z score analysis was used to evaluate the stabilization of the 12 modules (Table 2). The Z scores of nine modules (black, blue, brown, green, gray, purple, turquoise, red, and yellow) were observed to be > 5 with *p* < 0.05. This result expressed that the nine modules were stable. Correlation analysis was performed between each stable module and UC characterization, as shown in Fig. 4. For example, black, gray, and yellow modules were positively related to UC, whereas turquoise, green, and yellow modules were negatively correlated with UC.

**Fig. 5:**
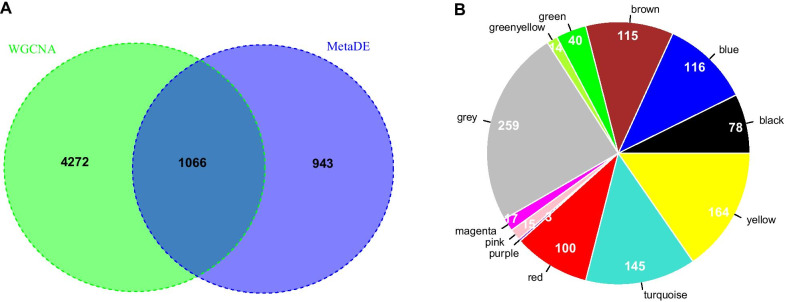
Comprehensive analysis of MetaDE and WGCNA. (**A**) The Venn diagram was compared based on the overlapping genes and the screening results of the MetaDE algorithm. (**B**) Based on the proportional pie chart of the gene number in the WGCNA algorithm stability module and the intersection genes screened by MetaDE in different color modules, each color corresponds to different color modules in the WGCNA, and the number indicates the gene number in the color module

A total of 1066 overlapping genes were screened through the comprehensive analysis of MetaDE and WGCNA (Fig. 5A; Table 2). Moreover, 78, 116, 115, 100, 145, and 164 overlapping genes from black, blue, brown, red, turquoise, and yellow modules were screened, respectively, whose fold enrichment were all > 1 and their p values were < 0.05 (Fig. 5B, Additional file 4: Table 4). Sixteen GO BPs and 12 KEGG pathways were screened, such as immune response, cytokine-cytokine receptor interaction, cell adhesion molecules (CAMs), and so on (Table 3).

**Table 4:** The significantly associated KEGG pathways with the feature genes obtained by machine learning

Term	Count	*p* Value
hsa04060:Cytokine-cytokine receptor interaction	24	6.840E−05
hsa04514:Cell adhesion molecules (CAMs)	15	2.900E−04
hsa04670:Leukocyte transendothelial migration	13	1.150E−03
hsa04062:Chemokine signaling pathway	17	1.214E−03
hsa04512:ECM-receptor interaction	10	3.326E−03
hsa04660:T cell receptor signaling pathway	11	5.800E−03
hsa04630:Jak-STAT signaling pathway	13	1.077E−02
hsa04650:Natural killer cell mediated cytotoxicity	11	2.308E−02
hsa05200:Pathways in cancer	20	2.981E−02

### Construction of the PPI network

**Fig. 6:**
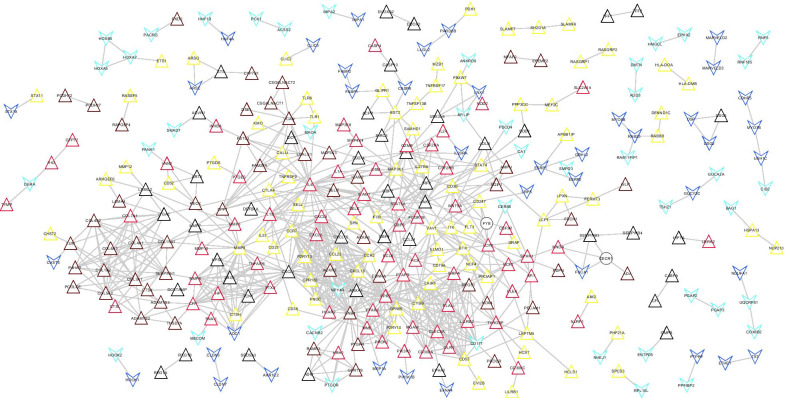
Construction of the PPI network. Triangles and inverted triangles represent the upregulated and downregulated genes in the tissues of patients with the disease, respectively. The color of the node indicates the corresponding color of the gene from the WGCNA module

The PPI network was established based on 718 overlapping genes. A total of 809 paired PPI interactions were obtained in this PPI network (Additional file 5: table 5). The network contained 329 nodes and 809 connection edges, as shown in Fig. 6. The KEGG pathway enrichment analysis was conducted on gene nodes that constitute the interaction network, and nine pathways with significant enrichment correlation were obtained, such as cytokine-cytokine receptor interaction, CAMs, leukocyte transendothelial migration, and so on (Table 4).

### Screening of important genes related to UC

A total of 157 KEGG pathways related to UC were selected by searching the UC database (Additional file 6: table 6). Nine overlapping pathways were obtained compared to KEGG pathways related to PPI interactions (Table 4), such as cytokine-cytokine receptor interaction, CAMs, leukocyte transendothelial migration, chemokine signaling pathway, extracellular matrix (ECM)-receptor interaction, T-cell receptor signaling pathway, Jak-STAT signaling pathway, natural killer cell-mediated cytotoxicity, and pathways in cancer. These KEGG pathways involved many genes involved in cytokine-cytokine receptor interaction (*CXCL1*, *CCR2*, *IL1B*, and *IL1A*), ECM-receptor interaction (*COL4A2*, *COL4A1*, *COL6A3*, *COL3A1*, and *COL1A2*), pathways in cancer (*STAT5A* and *SP11*), and leukocyte transendothelial migration and chemokine signaling pathway (*ITK*).

### Construction of the sample-type recognition classifier

**Fig. 7:**
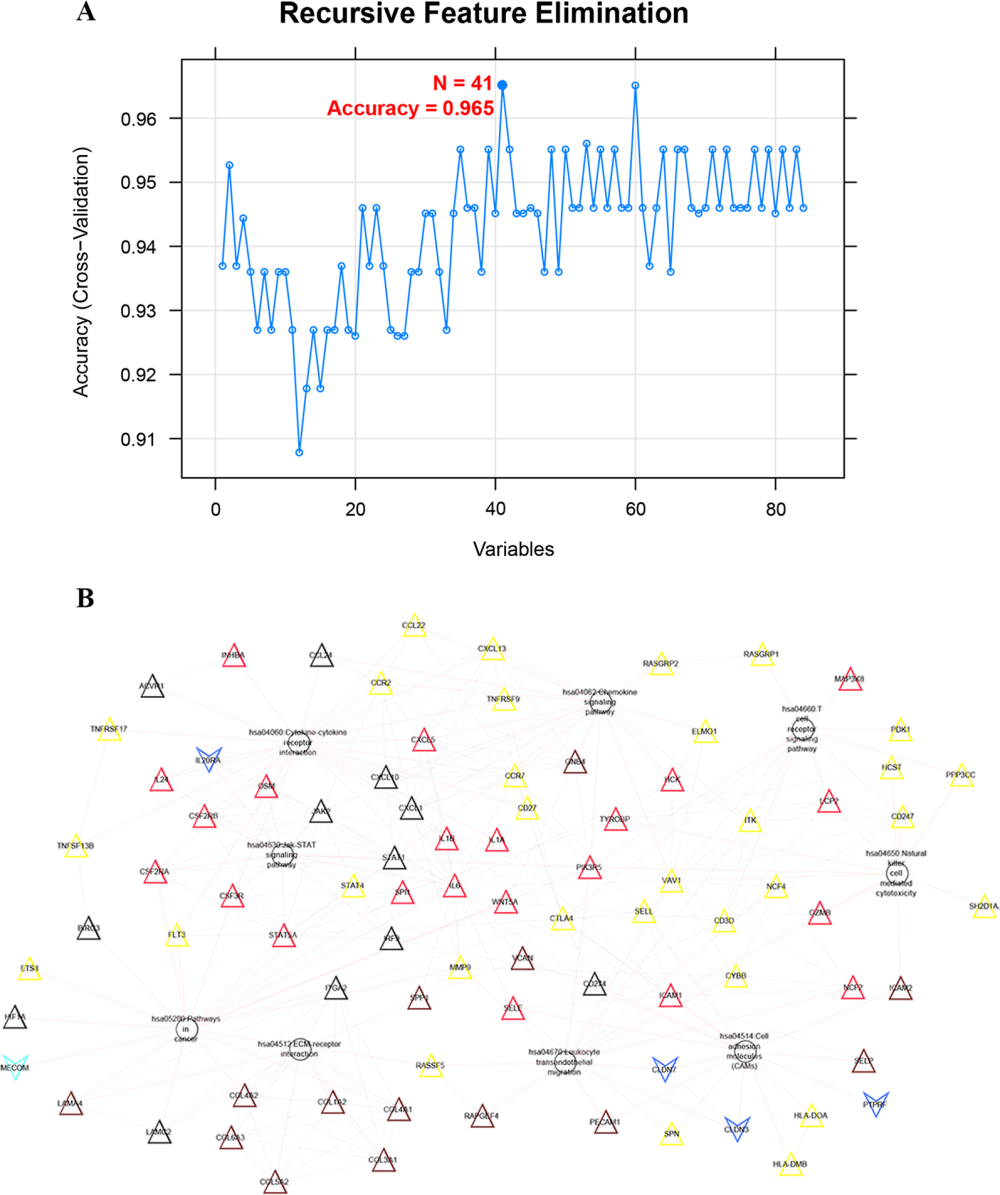
Feature genes were screened via RFE analysis

**Fig. 8:**
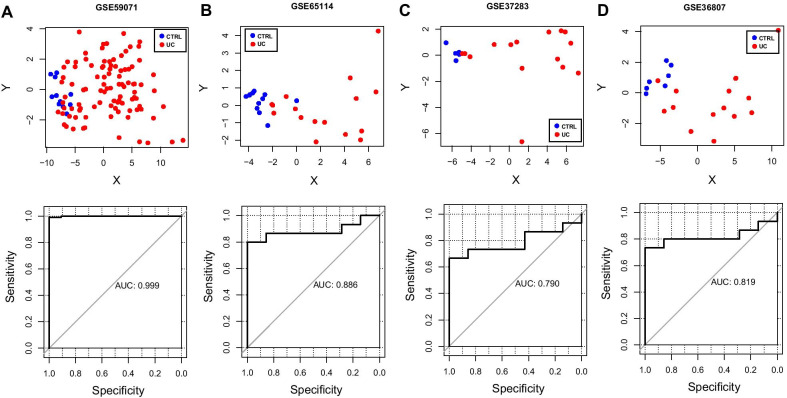
Binary scatter diagram of SVM for GSE59071 (A), GSE65114 (B), GSE37283 (C), and GSE36807 (D). Blue and red dots and squares represent the control and UC samples, respectively. The ROC curve of the SVM results of each dataset is shown in the figure below

A sample-type classifier was constructed based on 84 gene expression levels in the pathway network constructed in the GSE59071 training dataset. From the RFE analysis, when the number of genes is 41, it has the highest accuracy of 0.965, which was used to establish an SVM classifier, such as *CXCL1*, *CCR2*, *IL1B*, *IL1A*, *COL4A2*, *COL4A1*, *COL6A3*, *COL3A1*, *COL1A2*, *STAT5A*, *SP11*, and *ITK* (Fig. 7, Additional file 7: table 7)*.* Receiver operating characteristic (ROC) curves were applied to verify the efficacy of the SVM classifier (Fig. 8). The AUC was 0.999 in the GSE59071 dataset, whereas the AUC was 0.886, 0.790, and 0.819 in the validation dataset (GSE65114, GSE37283, and GSE36807, respectively).

## Discussion

In this study, 12 highly preserved modules were screened through the WGCNA. A PPI network was also constructed based on 718 overlapping genes. Besides, an SVM classifier combined with RFE was applied to explore the characteristic genes and pathways. From the RFE analysis, when the number of genes is 41, it has the highest accuracy of 0.965. The AUC was 0.999 in the training dataset, whereas the AUC was 0.886, 0.790, and 0.819 in the validation dataset (GSE65114, GSE37283, and GSE36807, respectively).

SVM is one of the most accurate methods among all well-known data mining algorithms. It is a two-class classification algorithm that can support linear and nonlinear classification. In this study, an SVM classifier was constructed to identify UC patients. Previous studies have reported that SVM could identify UC patients. For example, Ding et al. [24] used the SVM to classify healthy people or those with inactive colitis with a sensitivity of 83.5% and 97.1%, respectively. Watanabe et al. [25] used the SVM to evaluate the progress of UC-related neoplasms with an accuracy of 86.8% and 98.1%, respectively. Compared to previous studies, the SVM classifier constructed has better performance and potential applications.

An SVM classifier was established based on 41 genes involved in cytokine-cytokine receptor interaction (*CXCL1*, *CCR2*, *IL1B*, and *IL1A*), ECM-receptor interaction (*COL4A2*, *COL4A1*, *COL6A3*, *COL3A1*, and *COL1A2*), pathways in cancer (*STAT5A* and *SP11*), and leukocyte transendothelial migration and chemokine signaling pathway (*ITK*). Previous reports have indicated the vital role of gene-environment interaction in UC-related disease. Boshagh et al. [26] found that *CXCL1* is involved in the pathogenesis of UC. *CXCL1* may be used as a therapeutic target for UC, although more studies using human samples are required. *CCR2*, a chemokine receptor, may influence the body by maintaining and expanding chronic inflammation due to the timely removal or neutralization of invading agents. Pei et al. [27] found that PC3-secreted microprotein chemoattracted Ly6C monocytes in a CCR2-dependent manner by in situ chemotaxis and adoptive transfer experiments, which is an important molecule in UC. Previous studies have distinguished the interleukin-1 receptor antagonist gene allele 2 (IL-1RN*2) as a biomarker of susceptibility in UC [28]. Carter et al. [29] reported that IL-1RN*2 was related to decreased IL-1ra protein and *IL-1RN* mRNA levels in the colonic mucosa by determining the *IL1A* and *IL1B* genotypes. A previous study reported that somatic mutations and altered pathways are one of the reasons why UC turns into cancer [30]. Besides, Yan et al. [9] found that *COL6A3* referred to apoptosis, and the phosphatidylinositol 3-kinase/Akt pathway is associated with nonsilent recurrent somatic mutations in UC. *COL3A1* is a profibrogenic extracellular matrix gene. Wu [31] also indicated that *COL3A1* was upregulated at the active/chronic inflammatory stages. Genetic variants in the region are associated with UC. Besides, Stadnicki et al. [32] found that *ITK* is significantly decreased in UC compared to noninflammatory controls. This report suggested that the release of *ITK* during inflammation plays a role in UC. Although there are few studies focused on the association between *COL4A2*, *COL4A1*, *COL1A2*, and *SP11* and UC, this finding will provide a theoretical basis for future research on therapeutic targets for UC. This work expressed that *CXCL1*, *CCR2*, *IL1B*, *IL1A*, *COL4A2*, *COL4A1*, *COL6A3*, *COL3A1*, *COL1A2*, *STAT5A*, *SP11*, and *ITK* may be potential markers for UC.

The surgical management of UC remains a difficult challenge, depending on the patient’s status (whether urgent, emergent, or elective) [33]. However, the definition of the best timing and procedure for each patient is the key for the management of UC patients [34]. Thus, the finding of novel biomarkers is important for managing the time and procedure for UC patients. Lai et al. [35] indicated that six hub genes, including *CXCR2* and *CXCR1*, were regarded as potential biomarkers for the classification of UC. Similarly, this study also found 11 key biomarkers related to UC that might be helpful to determine the timing and procedure for UC patients. However, verification of these hub genes would need further experiments that involve UC patient samples.

## Conclusions

An SVM classifier based on feature genes could accurately identify healthy people or UC patients. This study may provide new insights into the molecular mechanism of UC.

## Supplementary Information

**Additional file 1.** Standardized expression level files of GSE26387, GSE26440, GSE13904 and GSE4607.

**Additional file 2.** Screening of 2009 differentially expressed genes with significant consistencywith significant consistency using MetaDE package.

**Additional file 3.** The genes in the nine significant stable modules, respectively.

**Additional file 4.** Significantly enriched genetic information in stable modules.

**Additional file 5.** A total of 809 pairs of interacting connected pairs in PPI network.

**Additional file 6.** A total of 157 KEGG pathways related to ulcerative colitis in Comparative Toxicogenomics Database database.

**Additional file 7.** Screening of optimized gene combinations using recursive feature elimination method.

## Data Availability

All data generated or analyzed during this study are included in this published article and its supplementary information files.

## Abbreviations

UC: Ulcerative colitis
IBD: Inflammatory bowel disease
WGCNA: Weighted gene co-expression network
DEGs: Differentially expressed genes
PPI: Protein–protein interaction
SVM: Support vector machine
RFE: Recursive feature elimination
NCBI: National Center for Biotechnology Information
GEO: Gene expression omnibus database
SMR: Standardized mean rank
CAMs: Cell adhesion molecules
UC: Ulcerative colitis
PRR: Pattern-recognition receptor
ITK: Intestinal tissue kallikrein
GO: Gene Ontology
BPs: Biological processes
KEGG: Kyoto encyclopedia of genes and genomes
FC: Fold change
